# Phloretin protects against cardiac damage and remodeling via restoring SIRT1 and anti-inflammatory effects in the streptozotocin-induced diabetic mouse model

**DOI:** 10.18632/aging.101954

**Published:** 2019-05-10

**Authors:** Yin Ying, Cheng Jiang, Meiling Zhang, Jiye Jin, Shuyu Ge, Xiaodong Wang

**Affiliations:** 1 Department of Pharmacy, Tongde Hospital of Zhejiang Province, Hangzhou 310012, Zhejiang, China; 2 Department of Rehabilitation, Tongde Hospital of Zhejiang Province, Hangzhou 310012, Zhejiang, China; 3 Department of Vascular Surgery, Tongde Hospital of Zhejiang Province, Hangzhou 310012, Zhejiang, China; * Equal contribution

**Keywords:** phloretin, anti-inflammation, SIRT1, diabetic cardiomyopathy

## Abstract

Diabetic cardiomyopathy increases the risk of heart failure independent of coronary artery disease and hypertension. Phloretin (PHL) shows anti-inflammatory effects in macrophages. In this study, we explored the protective effects of PHL on high glucose (HG)-induced injury in diabetic cardiomyopathy *in vivo* and *in vitro*. Using streptozotocin-induced diabetic mouse model and incubating cardiac cells line under a HG environment, PHL were evaluated of the activities of anti-inflammation and anti-fibrosis. In the study, PHL treatment ameliorated cardiomyocyte inflammation injury, and reduced fibrosis *in vivo* and *in vitro*. PHL also improved cardiac biochemical criterions after 8 weeks of induction of diabetes in C57BL/6 mice. Molecular docking results indicated that silent information regulator 2 homolog 1 (SIRT1) bound to PHL directly and that SIRT1 expression was upregulated in the PHL-treated group in HG-induced H9C2 cells. Protective effect of PHL was been eliminated in silence SIRT1 H9C2 cells. Taken together, these results suggested that PHL suppressed HG-induced cardiomyocyte injury via restoring SIRT1 expression.

## Introduction

It is now widely accepted that diabetes mellitus poses a severe threat to human health [1]. As one of the most frequently occurring vascular complications of diabetes, diabetic cardiomyopathy (DCM) exerts direct adverse effects on heart tissue. At its terminal stage, DCM can even result in heart failure [2]. The underlying pathophysiological mechanism involves abnormal activation in cardiomyocytes associated with abnormal cellular metabolism, chronic inflammation and fibrosis. These changes may lead to abnormalities in heart function and vascular dynamics [3]. The morbidity and mortality associated with these diseases mean that it is essential to develop drugs that can effectively delay myocardial remodeling and treat diabetic cardiac disease. Recent evidence indicates that inflammation is closely related to DCM. Hyperglycemia induces the inflammatory cascade and promotes the development and progression of DCM *in vivo*. In a nutshell, the mechanism underlying in DCM has been increasingly recognized.

**Figure 1:**
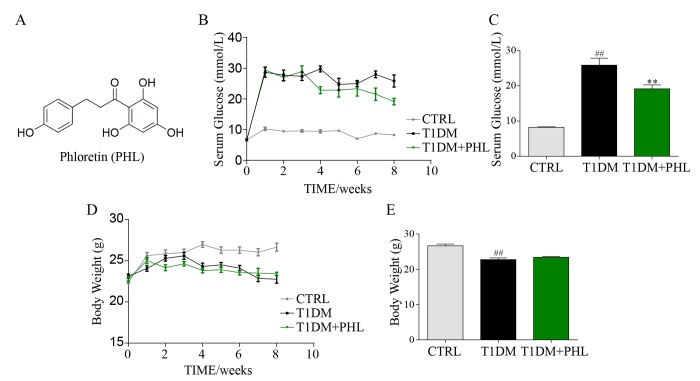
**Phloretin decreased blood glucose concentrations, but did not affect body weight.** The chemical structure of phloretin (PHL, **A**). Diabetes mellitus was induced in male C57BL/6 mice by a single intraperitoneal (i.p.) injection of STZ and mice with fasting-blood glucose > 12 mM were considered as diabetes and then diabetic mice were orally treated with PHL (20 mg/kg), or vehicle by gavage once every two days for 8 weeks (n =8 in each group), which was administrated in diabetic mice. In this process, the blood glucose (**B**-**C**) and body weight (**D**-**E**) were monitored once every week. **P < 0.01 v.s. T1DM group; ^##^P < 0.01 v.s. CTRL group.

A number of natural flavonoid products with anti-inflammatory effects have been tested to see whether they could serve as effective and safe therapies for DCM [4–6]. Phloretin (PHL, Figure 1A), a compound isolated from apple and pear trees, has been reported to have excellent anti-inflammatory activity [7]. Many studies have shown that it can exert multiple beneficial effects on cardiovascular performance, including alleviating sepsis in rats, increasing lipolysis in adipocytes [8], regulating glucose transporter function and inhibiting lipopolysaccharide (LPS)-induced release of inflammatory cytokines in macrophages and dendritic cells [9–13].

A growing number of studies have shown that flavonoids improves glucose-induced of diabetic complications via sirtuin family especially Silent information regulator 2 homolog 1 (SIRT1) [4,14–16]. SIRT1 is a member of the sirtuin family of class III histone deacetylases [17]. Sirtuins regulate important metabolic pathways in prokaryotes and eukaryotes, and are involved in many biological processes, including cell survival, proliferation, cell metabolism and calorie restriction [18]. SIRT1 is found in the nucleus and cytoplasm, and is considered to be a potential target for treatment of human pathologies, including cardiovascular disease, like diabetic cardiomyopathy [19]. Continuing research into the mechanisms underlying SIRT1-based treatment is necessary to further improve the efficacy and specificity of drugs against diabetic cardiomyopathy.

Thus, we investigated the protective effect of PHL against high glucose (HG)-induced DCM and further clarified the underlying molecular mechanisms. During this pre-experimentation, we showed that PHL suppressed inflammatory and fibrotic reactions by inhibiting expression of collagen I (COL-1A1), transforming growth factor (TGF-β) and inhibitor of NF-κB (IκB) in cardiac cell line. Then, we found that SIRT1 expression was upregulated when PHL (10μM) pre-treatment in rat myocardium myoblast (H9C2) cells. Further, molecular docking results indicated that SIRT1 may be bound to PHL directly, which implies that PHL protected cardiac cells from hyperglycemia by targeting SIRT1. Targeting SIRT1 by PHL thus revealed an unprecedented mechanism underlying the biological action of PHL. It also shed light on the potential available of PHL in the treatment of DCM.

## RESULTS

### Phloretin decreased blood glucose concentrations but did not affect body weight

Two weeks after STZ, mice exhibited a significant increase in blood glucose concentrations (Figure 1B) that remained high over the subsequent eight weeks. After treatment of PHL (20 mg/kg) for 8 weeks, the hyperglycemia of type 1 diabetes (T1DM) decreased. However, PHL treatment group still had a higher hyperglycemia than CTRL group (Figure 1B-C). The diabetic mice had a relatively low body weight (Figure 1D) during the experiment. PHL (20 mg/kg) treatment for 8 weeks resulted in no significant difference in body weight (Figure 1D-E) concentrations between the diabetic mellitus and PHL-treated groups. These results indicate that PHL could slightly attenuate HG level but could not inhibit the development of T1DM.

### Phloretin prevented cardiomyocyte injury in diabetic mice.

**Figure 2:**
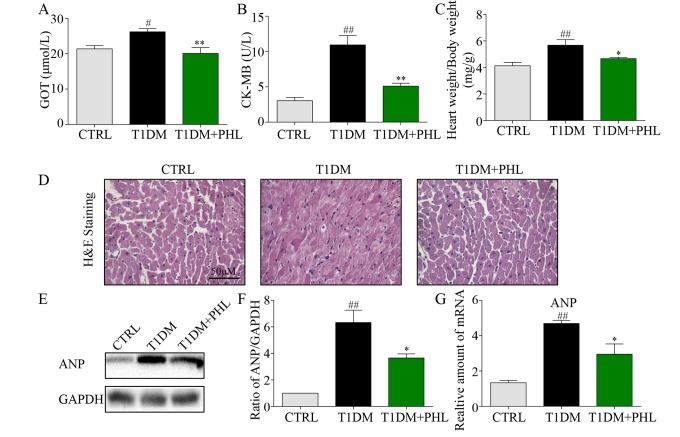
**Phloretin reduced hyperglycemia-induced biochemical indicators, improved histological abnormalities and cardiac hypertrophy of diabetic cardiomyopathy.** (**A**) The serum level of AST. (**B**) The serum level of CK-MB. (**C**) The ratio of heart weight/body weight. (**D**) Representative histomorphometric images for the haematoxylin and eosin staining (H&E) of formalin-fixed myocardial tissues from each group (400x magnification). (**E**-**F**) Western blot analysis for the protein expression of ANP in the myocardial tissues was performed. (**G**) The mRNA expression of the pro-hypertrophic gene ANP in myocardial tissues was detected by RT-qPCR. More than five mice in each group were used for above analysis. *P < 0.05, **P < 0.01 v.s. T1DM group; ^#^P < 0.05, ^##^P < 0.01 v.s. CTRL group.

DCM is commonly characterized by concentric hypertrophy, dilated cardiomyopathy, and extracellular fibrosis [20]. To evaluate whether PHL could protect diabetic mice from DCM, biochemical markers of myocardial injury including GOT, CK-MB, and heart weight/body weight were assessed. As shown in Figures 2A-2C, PHL significantly improved diabetes-induced increases in GOT, CK-MB and heart weight. H&E staining revealed disorganized myofibers and deranged cellular structures in the heart tissues of diabetic mice, while these pathological changes were reduced in mice treated with PHL (Figure 2D). To further evaluate heart pathologic changes, the atrial natriuretic peptide (ANP) protein and mRNA level were tested, with PHL shown to reduce ANP upregulation caused by hyperglycemia (Figures 2E-2G). These results suggested that PHL could restrain the pathological changes in heart tissues.

### Phloretin attenuated hyperglycemia-induced fibrotic response and apoptotic death in cardiomyocytes

**Figure 3:**
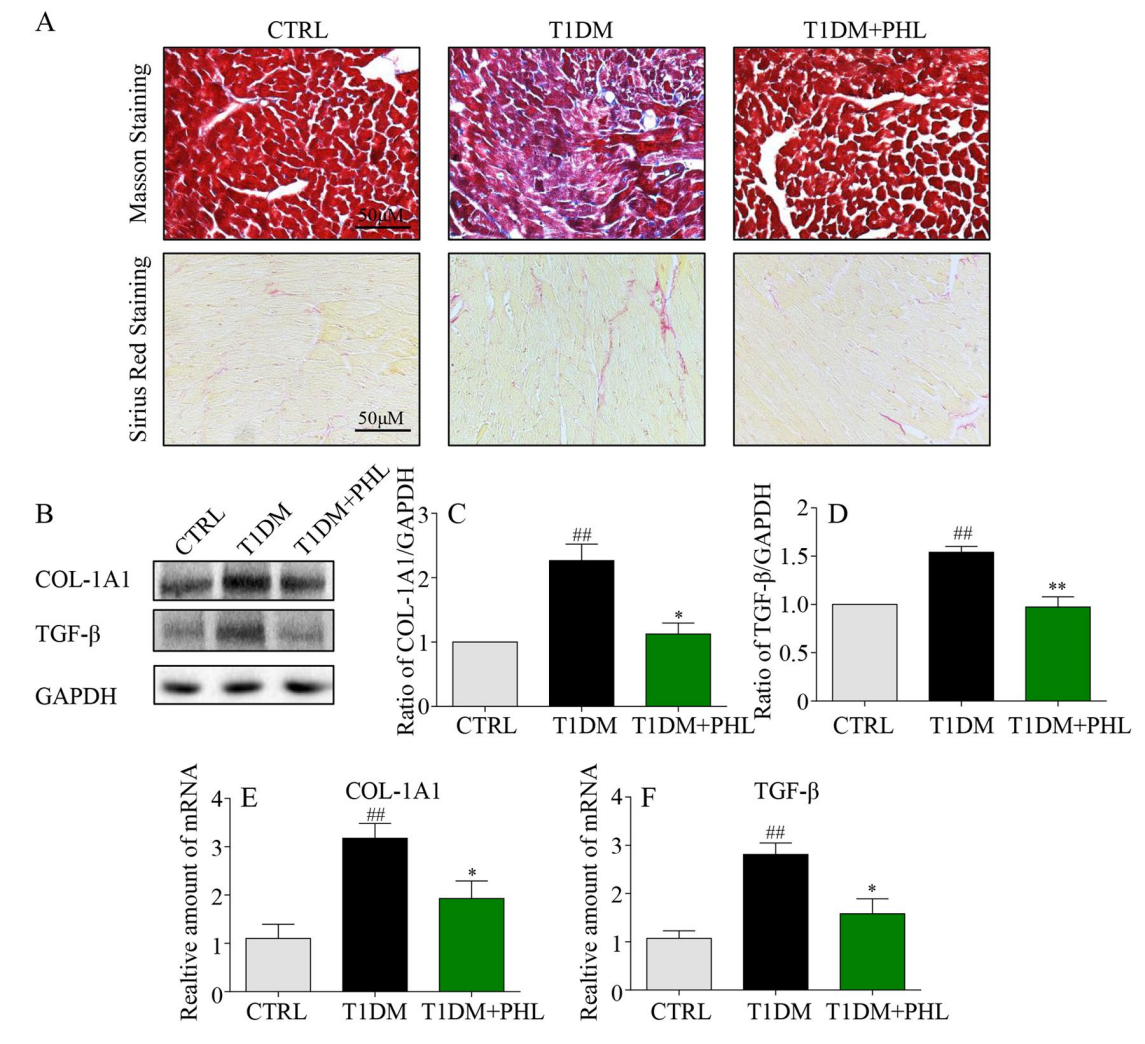
**Phloretin reduced hyperglycemia-induced fibrosis in the heart of diabetic mice.** (**A**) Myocardial tissues from each group were subjected to Masson’s Trichrome staining and Sirius red staining assay as described in Methods. (**B**-**C**) Western blot analysis for the protein expression of COL-1A1 and TGF-β in the myocardial tissues, with GAPDH as a loading control. (**D**-**F**) The mRNA expression levels of COL-1A1 and TGF-β in myocardial tissues of each group were determined by real-time qPCR. More than five mice in each group were used for above analysis. *P < 0.05, **P < 0.01 v.s. T1DM group; ^#^P < 0.05, ^##^P < 0.01 v.s. CTRL group.

Myocardial fibrosis is a major mechanism contributing to cardiomyocyte changes in diabetic cardiomyopathy [21,22]. Hyperglycemia induces formation of structural proteins and type 1 collagens, and promotes interstitial fibrosis and myocardial stiffness to produce subsequent heart failure [20]. Masson’s trichrome and sirius red staining are two typical methods to determine the fibrosis in tissues [23,24]. As shown in Figure 3A, increased COL-1A1 and fibrosis were observed in diabetic hearts, while these fibrotic changes were significantly reduced in the PHL-treated groups. Western blot analysis revealed a significant increase in pro-fibrotic TGF-β and fibrotic COL-1A1 genes expression in diabetic mouse cardiac tissues (Figures 3B-3D). Similar results were also observed in mRNA levels (Figure 3E-3F). Accordingly, hyperglycemia-induced expressions of COL-1A1 and TGF-β were significantly suppressed by PHL in diabetic mice. Thus, PHL attenuated hyperglycemia-induced fibrotic response in cardiomyocytes.

### Phloretin inhibited cardiomyocyte inflammatory response induced by diabetes

**Figure 4:**
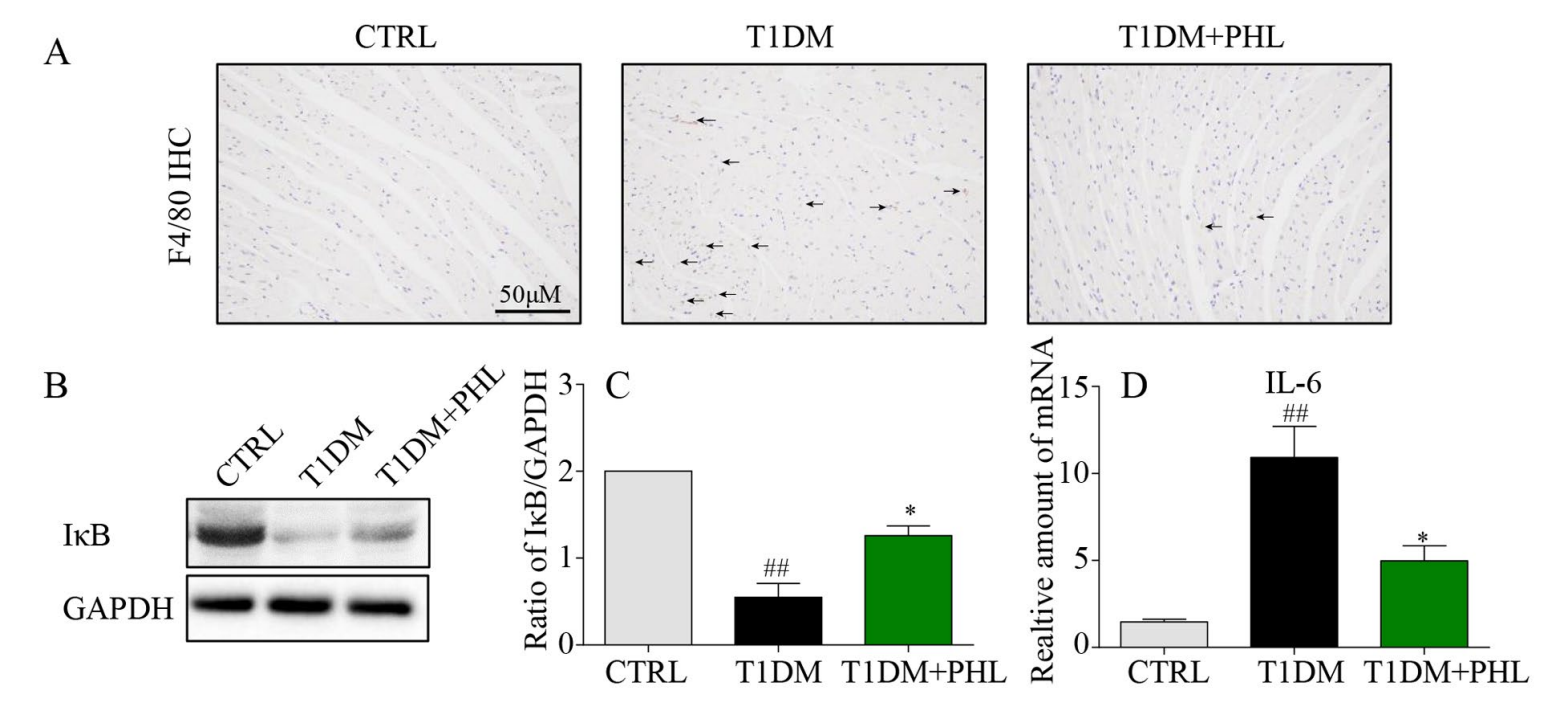
**Phloretin reduced hyperglycemia-induced inflammation in the heart of diabetic mice.** (**A**) Heart tissues were subjected to IHC analysis with anti-F4/80 antibodies as described in Methods (400x magnification). (**B**-**C**) Western blot analysis for the IκB degradation in the myocardial tissues, with GAPDH as a loading control. (**D**) The mRNA expression levels of IL-6 in myocardial tissues of each group were determined by real-time qPCR. More than five mice in each group were used for above analysis. *P < 0.05, **P < 0.01 v.s. T1DM group; ^#^P < 0.05, ^##^P < 0.01 v.s. CTRL group.

Hyperglycemia stimulates cardiomyocyte inflammatory processes, including macrophage infiltration activation and controlling inflammatory gene IκB degradation, leading to cardiomyocyte injury [25–27]. Therefore, we determined the effects of PHL on macrophage infiltration and inflammatory activity in heart tissues from diabetic, CTRL and PHL-treated mice. The immunofluorescence staining of F4/80 confirmed increased macrophage infiltration in heart tissue from diabetic mice (Figure 4A), and that this was suppressed in PHL-treated mice. Additionally, protein levels of IκB (Figures 4B-4C) were upregulated in diabetic mice compared with control mice, and the upregulation was decreased by PHL. Then, the mRNA expression of pro-inflammatory cytokines, IL-6, was determined (Figure 4D). PHL also exhibited excellent inhibition against HG-induced mRNA expression of this cytokines. Thus, PHL showed the protection activity in cardiomyocytes from inflammatory injury caused by hyperglycemia.

### Phloretin inhibited HG-induced inflammation and fibrosis via restoring of SIRT1 in H9C2

**Figure 5:**
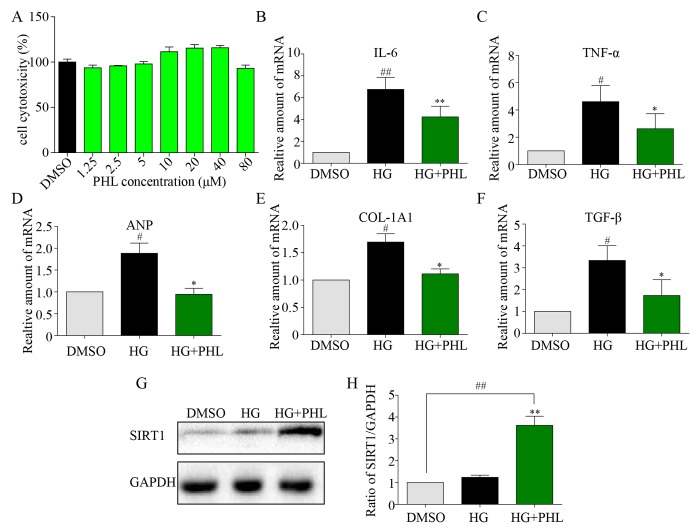
**Phloretin restoring SIRT1 to inhibit hyperglycemia-induced inflammation and fibrosis in H9C2 cells.** (**A**) The cytotoxicity of PHL in H9C2 cell. (**B**-**C**) H9C2 (1*10^6) cells were pre-treated with PHL (20 μM) for 1 h and then incubated with HG (33 mM) for 24 h. The cell lysates were immunoblotted for SIRT1, with GAPDH as a loading control. (**D**-**H**) H9C2 cells pre-treated with PHL for 1 h were stimulated by HG (33 mM). Total RNAs were extracted and the mRNA levels of IL-6 and TNF-α in 12 h (**D**-**E**) or ANP, COL-1A1 and TGF-β (**F**-**H**) in 36 h were detected by RT-qPCR. Data are presented as means ± SEM. *P < 0.05, **P < 0.01 v.s. HG group; ^#^P < 0.05, ^##^P < 0.01 v.s. DMSO group.

To further explore the mechanism underlying PHL’s protection of cardiomyocytes from HG-induced inflammatory and fibrotic injury, we chose the rat myocardium myoblast (H9C2) cell line to simulate the pathological process *in vitro*. Results from the MTT assay indicated no significant toxic effect of PHL on cells even when treated with six doses, even in 80 μM (Figure 5A). Firstly, inflammatory and hypertrophy cytokines were also assessed, with mRNA levels of IL-6, TNF-α and ANP significantly upregulated after 24 h of HG stimulation while upregulation was suppressed by PHL (20 μM) (Figures 5B-5D). The fibrotic mRNA transcription levels of COL-1A1 and TGF-βafter incubation with HG for 36 h were higher than those in the control group (Figure 5E-5F). Pre-treatment with PHL inhibited the change significantly.

**Figure 6:**
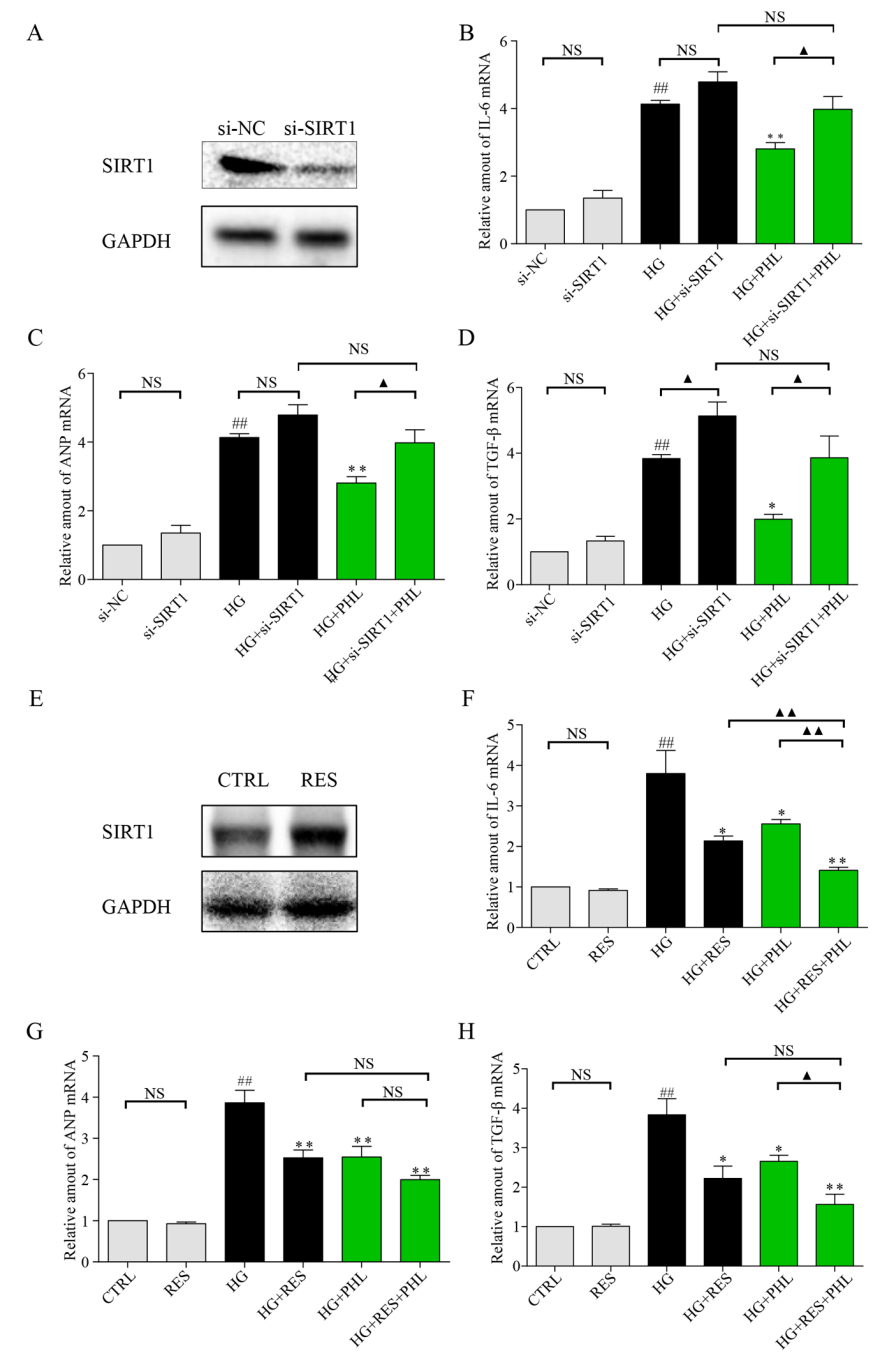
**Knockdown of SIRT1 aggravate HG induced inflammation, hypertrophy and fibrosis mRNA level in H9C2 cells.** (**A**) Knockdown of SIRT1 expression by siRNA in H9C2 cells. Assessment of SIRT1 protein levels was performed 48 h following transfection. (**B**-**D**) Real-time qPCR assay showed the mRNA levels of IL-6 (**B**), ANP (**C**), and TGF-β (**D**) in negative control (NC) or SIRT1 siRNA transfected H9C2 cells treated with HG. H9C2 cells pre-treated with PHL (20 μM) for 1 h were stimulated by HG (33 mM). (**E**) Overexpression of SIRT1 expression by RES (50 μM) in H9C2 cells. Assessment of SIRT1 protein levels was performed 24 h after treatment for RES. (**F**-**H**) H9C2 cells pre-treated with RES, PHL or RES+PHL for 1 h were stimulated by HG (33 mM). Real-time qPCR assay showed the mRNA levels of IL-6 (**F**), ANP (**G**), and TGF-β (**H**). Data are presented as means ± SEM. *P < 0.05, **P < 0.01 v.s. HG group; ^##^P < 0.01 v.s. DMSO group; and ^▲^P < 0.05, ^▲▲^P < 0.01, ‘NS’ means no significant.

As previous research indicates that SIRT1 is involved in HG-induced renal and retinal injury *in vivo* [28], and that flavonoid agents have the potential to restore of SIRT1 [4,6], we investigated H9C2 cells after pre-incubation with DMSO or PHL in HG stimulation. The results showed that SIRT1 expression increased only after stimulation by both PHL and HG (Figures 5G-5H). Next, we determined the effects of siRNA-mediated depletion of SIRT1 and resveratrol-mediated over-expression of SIRT1. As shown in Figure 6A, si-SIRT1 markedly attenuated SIRT1 expression in H9C2 cells. Creating SIRT1 deficit led to stronger effects of HG-induced expression of IL-6, ANP and TGF-β (Figure 6B-6D). Moreover, PHL protective function was significantly decreased after SIRT1 protein silence (Figure 6B-6D). Resveratrol (RES) is a common compound, which can activate SIRT1 expression [29]. As shown in Figure 6E, RES (50 μM) markedly activated SIRT1 expression in H9C2 cells. Compared with RES treatment group, PHL showed the same therapeutic effects in IL-6, ANP and TGF-β (Figure 6F-6H). Notably, RES and PHL combination showed greater inhibition to cure heart inflammation, hypertrophy and fibrosis (Figure 6F-6H).

### Molecular docking predicted the possible interaction between SIRT1 and PHL

**Figure 7:**
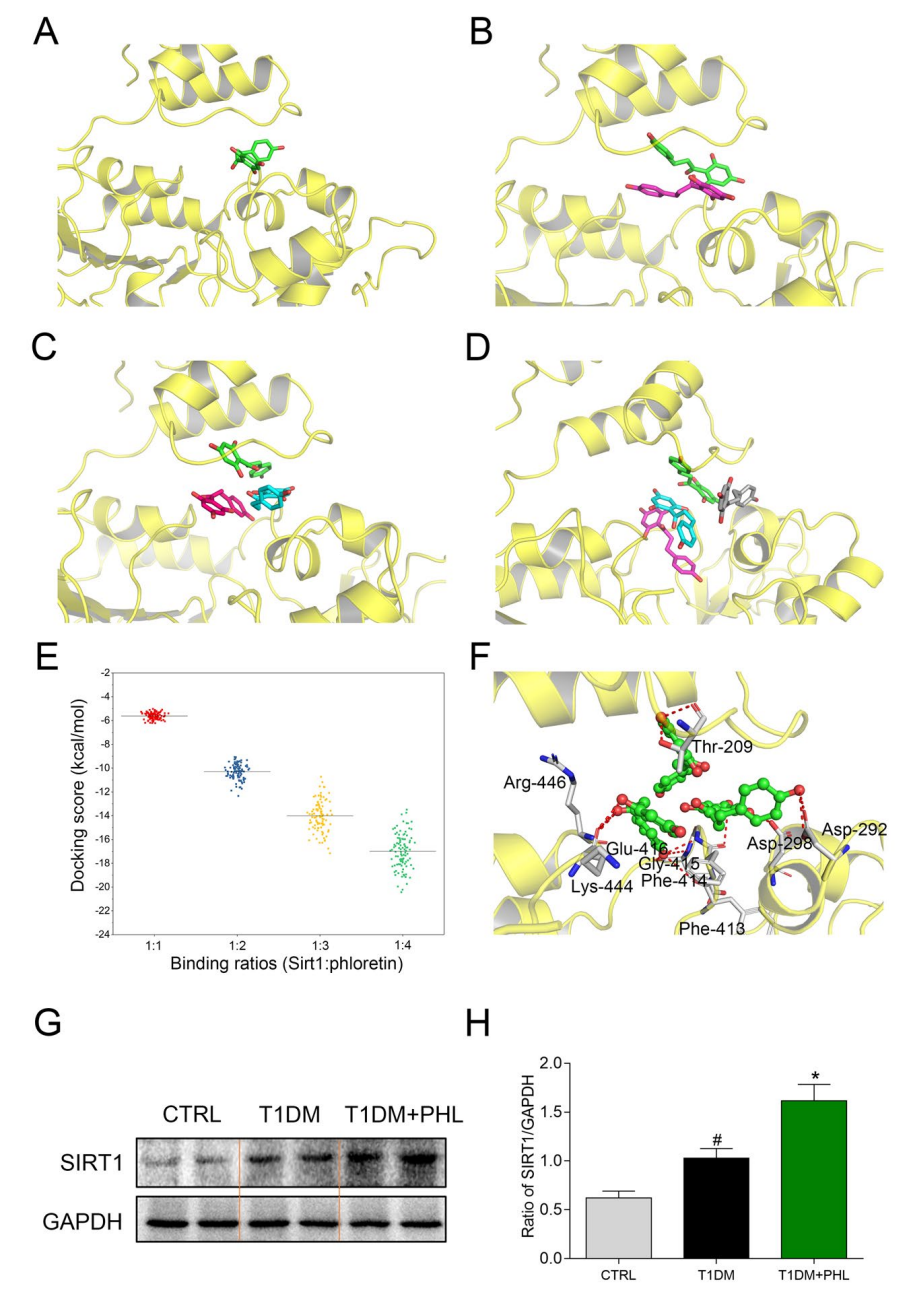
**Molecular docking analysis of PHL within binding site of SIRT1 at SIRT1: PHL binding ratios of 1:1, 1:2, 1:3, and 1:4.** (**A**) 1:1; (**B**) 1:2; (**C**) 1:3; (**D**) 1:4; (**E**) The distribution of 100 conformational scores for each ratio; (**F**) The detailed view of SIRT1: PHL with the binding ratio of 1:3. (**G**-**H**) Total proteins (100 μg) were extracted from the cardiac tissues in CTRL, T1DM and T1DM+PHL group. The expression level of SIRT1 in total protein was examined by western blot, with GAPDH as a loading control. Data are presented as means ± SEM. *P < 0.05 v.s. HG group; ^#^P < 0.05 v.s. DMSO group.

Molecular docking was used to predict the possible binding of PHL within the active cavity of SIRT1 at SIRT1:PHL binding ratios of 1:1, 1:2, 1:3 and 1:4 (Figure 7A-7D). Up to three PHL molecules bound within the binding pocket of SIRT1, which can be appropriately binding. With four PHL molecules, one of the molecules (colored in grey) presented with an unnatural binding pose, which seemed to be located outside the SIRT1 binding pocket. Next, the distribution of docking scores (free energy of binding) were analyzed. It was determined that an increase in binding ratio corresponded with higher binding affinity, with the lowest score at 1:4 binding ratios (Figure 7E). However, as the ratio of 1:4 yielded an unnatural binding conformation with SIRT1, we selected a ratio of 1:3 for further analysis. As shown in Figure 7F, residues of Thr-209, Asp-292, Asp-298, Phe-413, Phe-414, Gly-415, Glu-416, Lys-444 and Arg-446 formed hydrogen bonds with three molecules of PHL. Of these residues, Asp-292, Asp-298 and Lys-444 were also observed in the crystal structure of SIRT1/resveratrol complex with the 1:3 ratio, which interacted with resveratrol via hydrogen bonds [30]. The observed results indicated that it is likely that three molecules of PHL bind within the SIRT1 pocket to create a stable complex. As shown in 7G and 7H, PHL significantly increased the expression of SIRT1 in diabetes mice compared with CTRL or T1DM groups. Along with the in *vitro*, *in vivo* and molecular docking results, these findings led us to hypothesize that PHL prevented HG-induced inflammatory and fibrotic responses in H9C2 by targeting SIRT1 and upregulating its expression.

## DISCUSSION

Diabetic cardiomyopathy is a common complication of diabetes, usually causing patients decades of morbidity followed by severe outcomes, such as contractile dysfunction, concentric left ventricular hypertrophy, and dilated cardiomyopathy [1,20]. The metabolic disturbances of diabetes are often accompanied by local inflammation and myocardial fibrosis that eventually develop into myocardial tissue damage and heart failure [26,31,32]. Thus, it is important to identify new therapeutic targets for the cardiac cell inflammation and fibrosis that results from sustained hyperglycemia.

Many natural products have been studied to determine their impact on diabetic cardiomyopathy. Among these, PHL has been reported to have cardiac protective effects [33]. In this experiment, cardiac cells showed disorganized myocytes and myofibers 10 weeks after diabetes induction that was completely remitted by treatment with PHL for eight weeks. Heart weights in the PHL-treated group remained within the normal range. PHL administration also reduced the abnormal elevations in indices of cardiac injury. All of these findings supported a potential role for PHL in attenuating diabetic cardiomyopathy.

Among the beneficial anti-diabetic actions of PHL, we found it to effectively reconstruct cardiac muscular structure by reducing collagen 1 and fibrosis formation and decreasing pro-fibrotic cytokine expression. By suppressing expression of TGF-β, which has a central role in cardiac remodeling, PHL effectively protected cardiac cells from myocardial remodeling, and kept the diabetic heart from failing [34,35]. PHL has also been reported to have an excellent anti-inflammatory effect. It can eliminate inflammatory cytokine expression in endothelial cell response to stress stimulation [36,37]. Additionally, it alleviates cytokine production in asthmatic mice, holding promise as a potential agent for the treatment for age-related lung fibrosis [37,38]. In our experiment, we found that administration of PHL to diabetic mice for eight weeks significantly alleviated hyperglycemia-induced inflammation. The mechanisms for this protection involved correction of abnormal IκB activation, which is a primary regulator of inflammatory responses and is activated in glucose overload [39,40]. Phlortein also successfully normalized pro-inflammatory cytokines, including TNF-α and IL-6, which are activated by HG and contribute to recruitment of macrophages and other inflammatory cells [41,42]. Therefore, PHL has strong cardiac cell protective effects against hyperglycemia.

SIRT1 has been reported to exert important protective effects against aging, atherosclerosis, hypertrophic stresses and ischemia/reperfusion injury [43–45]. SIRT1 expression has been found to be significantly decreased in diabetic hearts, while resveratrol treatment could markedly restore SIRT1 expression with cardiac protective effects [46–48]. Fang and Ma showed that SIRT1 expression was reduced in a mouse model of diabetic cardiomyopathy resulting in both compromised insulin signaling and mitochondrial dynamic abnormity. Deletion of cardiac SIRT1 expression contributed to phenotypes resembling diabetic cardiomyopathy [49–51]. Our experiment confirmed that HG downregulated SIRT1 expression in H9C2 cells. We further showed that PHL upregulated SIRT1 expression in cardiomyocytes under HG conditions while in normal glucose cells, SIRT1 expression remained steady. PHL improved cardiac dysfunction, ameliorated cardiomyocyte fibrosis and attenuated inflammation injury in H9C2 cells under hyperglycemic conditions. After silencing of SIRT1, the protective role of PHL was significantly reduced. This surprising finding suggested that PHL may implement its cardiac protective effects through upregulation of SIRT1. Notably, diabetes mainly divides into two types about type 1 (T1DM) and type 2 (T2DM). Compared with T1DM, T2DM has a closer relationship with SIRT1. Thus, PHL might have some protective activation in T2DM. However; the mechanism underlying PHL activation remains obscure and requires continued research in the future.

In conclusion, we found that PHL could protect cardiomyocytes against HG-induced inflammation and fibrosis in H9C2 cell lines, with low cell cytotoxicity. We further found that it relieved abnormal increases in inflammatory cytokine mRNA transformation and reduced fibrosis. PHL probably exerted its cardioprotective effects via promotion of SIRT1 expression in the HG environment. Considering that SIRT1 has multiple functions [44,49], we speculated that activation of SIRT1 may provide beneficial effects beyond the heart that was tested here. PHL is a promising natural product that could protect against diabetic cardiomyopathy with its therapeutic target being SIRT1. Further studies are needed to explore whether SIRT1 is the only target of PHL. However, based on the present study, PHL has potential to be developed as a therapeutic medicine targeting diabetic cardiomyopathy.

## MATERIALS AND METHODS

### Reagents

Phloretin (PHL, PubChem CID: 24278652) and resveratrol (RES, PubChem CID: 445154), with a purity of 98%, was purchased from Aladdin (Shanghai, China). PHL and RES was dissolved in 0.5% sodium carboxyl methyl cellulose (CMC-Na) for *in vivo* experiments and in dimethyl sulfoxide (DMSO) for *in vitro* experiments [52,53]. Glucose and streptozotocin (STZ) were purchased from Sigma-Aldrich (St. Louis, MO, USA). Haematoxylin-eosin (H&E) was purchased from Beyotime (Nantong, China). Masson’s trichrome kits were obtained from Solarbio (Beijing, China). The assay kits for glutamic oxalacetic transaminase (GOT) and creatine kinase (CK-MB) were purchased from Nanjing Jiancheng Bioengineering Institute (Nanjing, China). Antibodies for TGF-β, collagen-1A1 (COL-1A1), IκB, glyceraldehyde-3-phosphate dehydrogenase (GAPDH) and secondary antibodies were purchased from Cell Signaling Technology (Danvers, USA). ANP and F4/80 were bought from Santa Cruz Technology (Santa Cruz, USA). RIPA lysis buffer was acquired from Boster Biological Technology (Wuhan, China).

### Animals and treatment

Twenty-four mice were purchased from Zhejiang Animal Center. The total mice were randomly divided into three groups. Sixteen mice received intraperitoneal (i.p.) injection of STZ 50 mg/kg, formulated in 100 mM citrate buffer (pH 4.5), each day for five consecutive days. Eight control animals received buffered saline only. Eight of the STZ-treated mice received PHL 20 mg/kg via i.g. seven days after commencing STZ. Blood glucose concentrations were measured in all 24 mice using a glucometer on days 7, 14, 21, 28, 35, 42, 49 and 56 after STZ induction. On day 56, the mice were killed under anesthesia and blood samples were drawn from the right ventricle using a heparinized syringe with a needle. After the mice died, their hearts were weighed. All experiments were performed strictly in accordance with the Provision and General Recommendation of Chinese Experimental Animals Administration Legislation.

### Determination of serum GOT and CK-MB

Serum concentrations of GOT and CK-MB were analyzed by commercial ELISA kits in accordance with the manufacturers’ instructions.

### Heart histopathology

Heart tissues were fixed in 4% paraformaldehyde and embedded in paraffin. The paraffin sections (5 μm) were stained with H&E, Masson’s trichrome and Sirius red. To estimate the extent of damage, specimens were observed under a light microscope.

### Immunohistochemistry for F4/80 detection

After deparaffinization and rehydration, 5 μm heart sections were treated with 3% H_2_O_2_ for 30 min and with 1% albumin from bovine serum (BSA) in phosphate buffered solution (PBS) for 30 min. Slides were incubated with anti-F4/80 antibody (1:100) at 4°C overnight and then with HRP-secondary antibody at room temperature for 1 h. Diaminobenzidine (DAB) was used for detection and hematoxylin to visualize nuclei. The images were viewed under fluorescence microscope.

### Cell culture

Rat myoblast cell line H9C2 was purchased from the Shanghai Institute of Biochemistry and Cell Biology (Shanghai, China). H9C2 was then cultured in DMEM medium with 1 g/L D-glucose, 10% FBS, 100 U/mL penicillin and 100 mg/mL streptomycin in a humidified atmosphere of 95% air and 5% CO_2_ at 37°C.

### Cell cytotoxicity

Cells were seeded into 96-well plates before treatment at 6,000 cells/well. PHL was added to the wells in various doses and incubated for 24 h. After treatment, MTT was added to each well (1 mg/mL) and incubated at 37°C for 4 h. Formazan crystals were dissolved with DMSO 150 μL/well. Absorbance was detected at 490 nm on a microplate reader. Cell cytotoxicity was expressed as the percentage of MTT reduction compared with control.

### Real-time quantitative PCR

Cells or heart tissues (20 mg) were homogenized in TRIZOL (Invitrogen, Carlsbad, CA) for extraction of RNA according to each manufacturer's protocol. Both reverse transcription and quantitative PCR were carried out using a two-step M-MLV Platinum SYBR Green qPCR SuperMix-UDG kit (Invitrogen, Carlsbad, CA). Eppendorf Mastercycler ep realplex detection system (Eppendorf, Hamburg, Germany) was used for q-PCR analysis. Gene primers including IL-6, TNF-α, ANP, COL-1A1, TGF-β and β-actin were obtained from Invitrogen (Shanghai, China). The primer sequences are listed in Table S1. The amount of each gene was determined and normalized to the amount of β-actin.

### Western blot analysis

Collected cells or homogenized heart tissue samples were lysed. Lysates were separated by 10% PAGE-1% SDS and electro transferred to a nitrocellulose membrane. Each membrane was pre-incubated in Tris-buffered saline (pH 7) containing 0.05% Tween 20 and 5% non-fat milk at room temperature for 1.5 h. The membrane was then incubated with specific antibodies at 4°C overnight. Immunoreactive bands were detected by incubating with secondary antibody conjugated with horseradish peroxidase and visualizing using enhanced chemiluminescence reagents. The amounts of the proteins were analyzed using Image J analysis software and normalized to GAPDH.

### RNA interference (siRNA)

si-SIRT1 and si-NC duplexes were purchased from Genepharma (Shanghai, China) and have the following sequences: Rat SIRT1 (forward 5′-GAAGUUGACCUCCUCAUUGUdTdT-3′ and reverse 5′-ACAAUGAGGAGGUCAACUUCdTdT-3′) using lipofectamine 2000 according to manufacturer's protocol.

### Molecular docking analysis

The binding poses of PHL in the active site of human SIRT1 was predicted by using the MLSD program [54]. The structure of SIRT1 was obtained from the Protein Data Bank (PDB) database (PDB code: 5BTR) [30]. The structure of SIRT1 was preprocessed by the UCSF ChimeraX software, including the removal of all the water molecules, hetero-atoms [55]. A grid box size of 22.5 Å× 22.5 Å× 22.5 Å was implemented and covered almost the entire binding site of SIRT1. Then different ratio of PHL was docked in to the active site of SIRT1 and 100 poses for each ratio were generated. The binding poses were scored by the particle swarm optimization (PSO) method, and then ranked by their docking energies. The lowest binding energy poses was considered for further analysis.

### Statistical analysis

The results are presented as means ± SEM. The statistical significance of differences between groups was obtained using one-way analysis of variance (ANOVA) for multiple comparisons in GraphPad Prism 7.0. Differences were considered to be significant at P<0.05.

## Supplementary Material

Supplementary Table
