# Supplementary material for: Phloretin protects against cardiac damage and remodeling via restoring SIRT1 and anti-inflammatory effects in the streptozotocin-induced diabetic mouse model
Source: Aging (Albany NY). 2019 May 10;11(9):2822–35. doi: 10.18632/aging.101954 (PMC6535073; doi:10.18632/aging.101954)
Supplement: Supplementary Table [file aging-11-101954-s001.pdf]

SUPPLEMENTARY TABLE

Table S1. Primer sequences of genes in RT-qPCR assay.

Gene	Species	Forward Primer	Reversed Primer
IL-6	Mouse	GAGGATACCACTCCCAACAGACC	AAGTGCATCATCGTTGTTCATACA
COL-1A1	Mouse	TGGCCTTGGAGGAAACTTTG	CTTGGAAACCTTGTGGACCAG
TGF-β	Mouse	TGACGTCACTGGAGTTGTACGG	GGTTCATGTCATGGATGGTGC
ANP	Mouse	AACCTGCTAGACCACCTGGA	TGCTTTTCAAGAGGGCAGAT
β-actin	Mouse	CCGTGAAAAGATGACCCAGA	TACGACCAGAGGCATACAG
IL-6	Rat	GAGTTGTGCAATGGCAATTC	ACTCCAGAAGACCAGAGCAG
TNF-α	Rat	TACTCCCAGGTTCTCTTCAAGG	GGAGGCTGACTTTCTCCTGGTA
COL-1A1	Rat	GAGCGGAGAGTACTGGATCGA	CTGACCTGTCTCCATGTTGCA
TGF-β	Rat	GGACTACTA CGCCAAAGAAG	TCAAAAGACAGCCACTCAGG
ANP	Rat	CTGCTAGACCACCTGGAGGA	AAGCTGTTGCAGCCTAGTCC
β-actin	Rat	ATCGTGGGCCGCCCTAGGCACC	CTCTTTAATGTCACGCACGATTTC

Supplementary Table 2. Statistical analysis for the N-acyl ethanolamines (NAEs) identified in the top lipid differences in abundance between the mouse and bat mitochondria.

Lipid	Comparison between	Statistical test	*p* value
NAE 18:2	BB/YMB/OMB	Kruskal-Wallis and Bonferroni correction	<0.0001*
	YMB/OMB	Mann-Whitney	0.0039*
	BB/YMB	Mann-Whitney	<0.0001*
	BB/OMB	Mann-Whitney	<0.0001*
NAE 20:4	BB/YMB/OMB	Kruskal-Wallis and Bonferroni correction	<0.0001*
	YMB/OMB	Mann-Whitney	0.0892
	BB/YMB	Mann-Whitney	<0.0001*
	BB/OMB	Mann-Whitney	<0.0001*
	BM/YMM/OMM	Kruskal-Wallis and Bonferroni correction	<0.0001*

m/z	RT (mins)	Lipid tentative identification	Fatty acid group	Fold change	Score
357.280	1.69	C24:5	PUFA	8.35	2
331.264	1.57	C22:4	PUFA	5.65	2
269.249	1.73	C17:0 Heptadecanoic acid	SFA	5.27	2
281.248	1.61	C18:1 Oleic	MUFA	4.87	2
303.233	1.32	C20:4 Arachadonic acid	PUFA	2.65	2
327.233	1.21	C22:6 Docosahexaenoic acid	PUFA	2.57	2
255.233	1.55	C16:0 Palmitic	SFA	2.75	2
277.217	1.17	C18:3 Linolenic	PUFA	11.13	2
225.186	0.96	C14:1	MUFA	4.78	2
317.248	1.45	C21:5	PUFA	1192.67	2
241.217	1.37	C15:0 Pentadecanoic acid	SFA	3.96	2
339.326	3.20	C22:0	SFA	2.28	2
295.227	0.61	C18 H31 O3	HFA	4.09	2
293.248	1.51	C19:2	PUFA	271.46	2
293.212	0.71	C18 H29 O3	HFA	567.49	2
367.358	4.03	C24:0	SFA	3.22	2
311.295	2.50	C20:1	MUFA	2.13	2
361.311	2.27	C24:3	PUFA	16.25	2
363.327	2.65	C24:2	PUFA	18.28	2
323.295	2.27	C21:1	MUFA	15.43	2
393.373	4.01	C26:1	MUFA	12.27	2
359.295	1.92	C24:4	PUFA	5.87	2
239.201	1.15	C15:1	MUFA	8.64	2
385.311	2.05	C26:5	PUFA	186.51	2
355.264	1.47	C24:6	PUFA	3.22	2
387.327	2.35	C26:4	PUFA	10.27	2
395.389	4.93	C26:0	SFA	2.92	2
325.311	2.84	C21:0	SFA	2.98	2
199.170	0.88	C12 SFFA	SFA	1.57	2
337.236	1.53	C22:1	MUFA	1.69	2
227.201	1.21	C14:0	SFA	1.40	2
391.358	3.34	C26:2	PUFA	30.76	2
313.078	3.28	C20:0	SFA	3.06	2

Odd carbon number fatty acids are highlighted since they are considered unusual but may be connected with health measures [4]. Lipids were identified using the human metabolome and Lipid maps databases. Abbreviations: Retention time (RT) in minutes, electrospray ionisation mode (ESI), polyunsaturated fatty acid (PUFA), saturated fatty acid (SFA), monounsaturated fatty acid (MUFA) and hydroxy faty acid (HFA). All ions are in negative mode. Scores according to Sumner et al., 2007 [2].

Supplementary Table 4. The fatty acids identified in the bat and mouse skeletal muscle mitochondria.

m/z	RT	Tentative lipid Identity	Fatty acid group	Increased expression in	Fold change	Score
293.212	0.71	C18 H29 O3	HFA	Bat	53.32	2
297.279	2.22	C19:0	SFA	Bat	23.78	2
295.227	0.61	C18 H31 O3	HFA	Bat	13.11	2
329.248	1.37	C22:5 Docosapentaenoic acid	PUFA	Bat	10.89	2
269.249	1.73	C17:0 Heptadecanoic acid	SFA	Bat	9.81	2
325.311	2.84	C21:0	SFA	Bat	6.03	2
337.311	2.54	C22:1	MUFA	Bat	5.74	2
327.233	1.21	C22:6 Docosahexaenoic acid	PUFA	Bat	4.70	2
303.233	1.32	C20:4 Arachadonic	PUFA	Bat	4.23	2

Supplementary Table 5. The fatty acids identified in the bat and mouse skeletal muscle mitochondria. Statistical analysis for the representative fatty acids presented in Figure 5 (D,E,F).

Lipid	Comparison between	Statistical test	*p* value
Docosahexaenoic acid	BB/YMB/OMB	Kruskal-Wallis and Bonferroni correction	<0.0001*
	YMB/OMB	Mann-Whitney	0.0052*
	BB/YMB	Mann-Whitney	<0.0001*
	BB/OMB	Mann-Whitney	<0.0001*
Arachidonic acid	BB/YMB/OMB	Kruskal-Wallis and Bonferroni correction	<0.0001*
	YMB/OMB	Mann-Whitney	<0.0001*
	BB/YMB	Mann-Whitney	<0.0001*
	BB/OMB	Mann-Whitney	<0.0001*
Docosapentaenoic acid	BM/YMM/OMM	Kruskal-Wallis and Bonferroni correction	<0.0001*
	YMM/OMM	Mann-Whitney	<0.0001*
	BM/YM	Mann-Whitney	<0.0001*
	BM/OMM	Mann-Whitney	<0.0001*

Number of samples; bat brain (BB) mitochondrial (adult, n=10), bat skeletal muscle (BM) mitochondria (adult, n=10), young mouse brain (YMB) mitochondria aged 4-11 weeks (n=10), old mouse brain mitochondria (OMB) aged 78 weeks (n=10), young mouse skeletal (YMM) muscle mitochondria aged 4-11 weeks (n=9) and aged mouse skeletal muscle mitochondria (OMM) aged 78 weeks (n=10). Statistical analysis was performed in GraphPad Prism.

357.280	1.69	C24:5	PUFA	Bat	1.64	2

Odd carbon number fatty acids are highlighted since they are considered unusual but may be connected with health measures [4]. Lipids were identified using the human metabolome and Lipid maps databases. Abbreviations: Retention time (RT) in minutes, electrospray ionisation mode (ESI), [illegible]

SUPPLEMENTARY REFERENCES

1. Shephard F, Greville-Heygate O, Marsh O, Anderson S, Chakrabarti L. A mitochondrial location for haemoglobins-Dynamic distribution in ageing and Parkinson’s disease. Mitochondrion. 2013; 14:64–72.

2. Sumner LW, et al. Proposed minimum reporting standards for chemical analysis Chemical Analysis Working Group (CAWG) Metabolomics Standards Initiative (MSI). Metabolomics. 2007; 3:211.

3. Gaffney CJ, Bass JJ, Barratt TF, Szewczyk NJ. Methods to assess subcellular compartments of muscle in C. elegans. J Vis Exp. 2014; 93:e52043.

4. Jenkins B, West JA, Koulman A. A review of odd-chain fatty acid metabolism and the role of pentadecanoic Acid (c15:0) and heptadecanoic Acid (c17:0) in health and disease. Molecules. 2015; 20:2425–44.
